# The impacts of climate change on women’s reproductive and sexual health: a systematic review

**DOI:** 10.1186/s12978-026-02375-0

**Published:** 2026-05-27

**Authors:** Muayad Saud Albadrani

**Affiliations:** 1 Department of Family and Community Medicine and Medical Education, College of Medicine, Taibah University, Madinah, 42353 Saudi Arabia; 2 Health and Life Research Center, Taibah University, Madinah, 42353 Saudi Arabia

**Keywords:** Climate change, Reproductive health, Sexual health, Systematic review

## Abstract

**Background:**

Climate change is considered a substantial threat to women’s health, particularly in their reproductive and sexual well-being. Several studies linked to changes in fertility rates, ovarian reserve, menopause timing, maternal and child health outcomes, and reproductive decision-making. While recent studies have suggested a correlation between climate variability and reproductive and sexual health outcomes, a comprehensive synthesis of existing research on this relationship is lacking.

**Aim:**

This study systematically reviews the available evidence on the association between climate change and women’s reproductive and sexual health.

**Methods:**

A thorough search was conducted across Medline/PubMed, Scopus, Web of Science, and Cochrane Library databases up to May 2024. This review follows a registered protocol with the PROSPERO database (ID: CRD42024575251). Data were extracted using a standardized template, and quality evaluation was carried out using the Joanna Briggs Institute’s Critical Appraisal Tools.

**Results:**

The search identified 3581 records, from which 12 observational studies were selected following screening. Most included studies were of moderate quality. Exposures were assessed using direct meteorological measures and subjective perceptions of climate change. The findings indicate that high temperatures are significantly associated with adverse outcomes. These include reduced ovarian reserve, lower fertility rates, and diminished reproductive decision-making. Extreme weather events were linked to negative social consequences like forced marriages, while specific regional climates were associated with premature menopause. Concerns about climate change also shape reproductive intentions, as eco-anxiety influences decisions to have smaller families. Conversely, higher latitude correlated with lower fertility rates.

**Conclusion:**

The review emphasizes the substantial adverse impacts of climate change, whether manifested through winter cold or rising temperatures, on women’s reproductive and sexual health. Enhanced public health strategies and more longitudinal studies are needed to establish causality and address women’s vulnerabilities in the face of escalating climate impact.

**Supplementary Information:**

The online version contains supplementary material available at 10.1186/s12978-026-02375-0.

## Introduction

Climate change is generally recognized as the most significant global challenge of the 21st century [1]. Global climate change is anticipated to yield notable demographic consequences for human societies. Environmental changes are increasingly recognized as important health risks for pregnant women, their offspring, and reproductive health [2]. Demography and epidemiology researchers have demonstrated that climate fluctuations significantly impact human death rates [3] and overall well-being [4].

Climate change significantly impacts women’s reproductive and sexual health (RSH) across various aspects. It is associated with underweight conditions and limited reproductive autonomy, although not necessarily affecting contraception usage [5]. There’s an observable trend of considering fewer children or exploring alternative paths, such as adoption, due to climate change concerns [6].

The most direct consequence of the change in the influence of worldwide climate on human beings is evident in the gradual rise in the average global heat, accompanied by heightened occurrences, severity, and duration of extreme heat events [7]. Higher temperatures correlate with diminished ovarian reserve and reduced fertility, raising concerns about accelerated reproductive aging [8]. Separately, extreme heat negatively impacts women’s capacity to function effectively, impeding their ability to attend to their own needs and those of their children, including breastfeeding practices [9]. These factors induced by climate intersect with socioeconomic circumstances, molding decisions related to RSH and behaviors concerning family planning.

The complex interconnection between climate change and RSH extends to encompass broader aspects of women’s and children’s health, underscoring vulnerabilities in areas marked by heightened risks associated with climate change [8].

The aim of this systematic review is to synthesize the evidence on the effects of climate change, including exposure to extreme temperatures and weather events, on the RSH of women of reproductive age. The review examined biological metrics such as fertility rates, ovarian reserve, and menopause timing. In addition, psychosocial dimensions, including reproductive decision-making and autonomy, were tackled.

While previous literature has not definitively established a connection between climate change and its influence on women’s reproductive function, this review seeks to address this knowledge gap and the discrepancies between different studies by thoroughly examining the significant effects of climate change on female RSH. Findings from this review may inform future research directions, guide theoretical frameworks on climate-health interactions, support clinical and educational interventions targeting RSH, and provide evidence for public health and policy planning.

## Methods

### Study design

This systematic review was conducted following the PRISMA guidelines and adhered to the protocol registered in the PROSPERO database (ID: CRD42024575251). The registration guarantees methodological transparency and supports adherence to systematic review best practices [9, 10].

### Search strategy

A comprehensive search strategy was designed and conducted to ensure thorough and unbiased coverage of the literature. The following databases were searched: Medline/PubMed, Scopus, Web of Science, and Cochrane Library. These databases were chosen based on their relevance and extensive coverage of medical, environmental, and public health literature. Grey literature was informally consulted, including policy documents from international organizations (e.g., World Health Organization [WHO], United Nations Population Fund [UNFPA], Intergovernmental Panel on Climate Change [IPCC]), as well as government health ministry websites and climate–health reports from selected countries, to gather supporting evidence that might be of use during discussion.

Search terms were developed using keywords and Medical Subject Headings (MeSH) related to climate change, reproductive health, and sexual health. Boolean operators (AND/OR) were applied to combine search terms, and the search strategy was pilot-tested for comprehensiveness against benchmark studies.

The full search strategy, including exact search terms and syntax for each database, is provided in the supplementary materials to ensure transparency and reproducibility (Supplementary Table 1).

### Criteria for eligibility

The Population (P) focused on women of reproductive age; the Exposure (E) included variables related to climate change, such as extreme temperatures and weather events; there was no Comparator (C), given the observational nature of the included studies; and the Outcomes (O) were defined as reproductive and sexual health metrics, including fertility rates, ovarian reserve, reproductive decision-making, and menopause timing. This framework informed the research question, search strategy, and inclusion criteria.

#### Inclusion criteria

This systematic review focused on research involving human participants, published exclusively in English, examining the influence of natural climate fluctuations on reproductive and sexual health. The review was inclusive of both studies measuring biological and physiological health outcomes (e.g., ovarian reserve, fertility rates, menopause timing) and those assessing psychosocial and behavioral outcomes (e.g., reproductive intentions, decision-making, preferences). All types of empirical study methodologies, including observational and survey studies, were considered, except literature reviews.

#### Exclusion criteria

Abstracts from conferences, studies not peer-reviewed, research involving plants or animals, and investigations utilizing artificial heat or cold sources. Furthermore, studies exclusively concerning pregnant women, those involving artificial reproductive techniques like In Vitro Fertilization (IVF), and research not specifically addressing women’s reproductive health were also excluded. Studies focusing exclusively on pregnant women were excluded because the present review specifically aimed to examine the impact of climate change on baseline reproductive and sexual health indicators in women of reproductive age (e.g., fertility, ovarian reserve, menopause timing, and reproductive decision-making), rather than maternal or obstetric outcomes during pregnancy. Pregnancy-specific outcomes (such as preterm birth, gestational complications, or birth weight) constitute a distinct body of literature with different exposure-response considerations and have already been the focus of several dedicated systematic reviews; their inclusion would therefore have broadened the scope beyond the predefined PECO framework.

### Screening procedure

Duplicates were removed using EndNote software (Clarivate Analytics, PA, USA). References were then assessed through two screening phases: the first involved evaluating the relevance of titles and abstracts against our eligibility criteria, while the second entailed a thorough examination of full-text articles selected from the initial screening to determine final eligibility for this review. Title and abstract screening, full-text eligibility assessment, data extraction, and quality appraisal were carried out independently by two reviewers, with any disagreements resolved through discussion and, where consensus could not be reached, by consultation with a third senior researcher who did not meet the authorship criteria; his role has been acknowledged accordingly. The Rayyan website [11] facilitated the process of selecting studies.

### Data gathering

A consistent data extraction template was utilized to systematically gather pertinent information from each study included. Extracted data included bibliographic information (author, publication year), study design, location, number of participants, inclusion criteria, measured outcomes, and key findings.

### Quality assessment

The quality of the included studies was assessed using the Joanna Briggs Institute (JBI) Critical Appraisal Tools, which are widely recognized and validated for systematic reviews of observational studies [12]. These tools evaluate potential bias and study quality across various domains, such as study design, data collection, and outcome reporting. The choice to use the JBI tools was based on their relevance and applicability to the diverse study designs included in this review. Detailed results of the critical appraisal, including individual study assessments, are provided in the supplementary materials to ensure transparency and rigor [12]. These tools comprise a set of inquiries, with scores indicating the study’s effectiveness in addressing potential bias areas [12]. Studies scoring 9 or above out of 11 for cohort studies, and 6 or above out of 8 for analytical cross-sectional studies were classified as high-quality.

## Results

### Literature search results

**Fig. 1:**
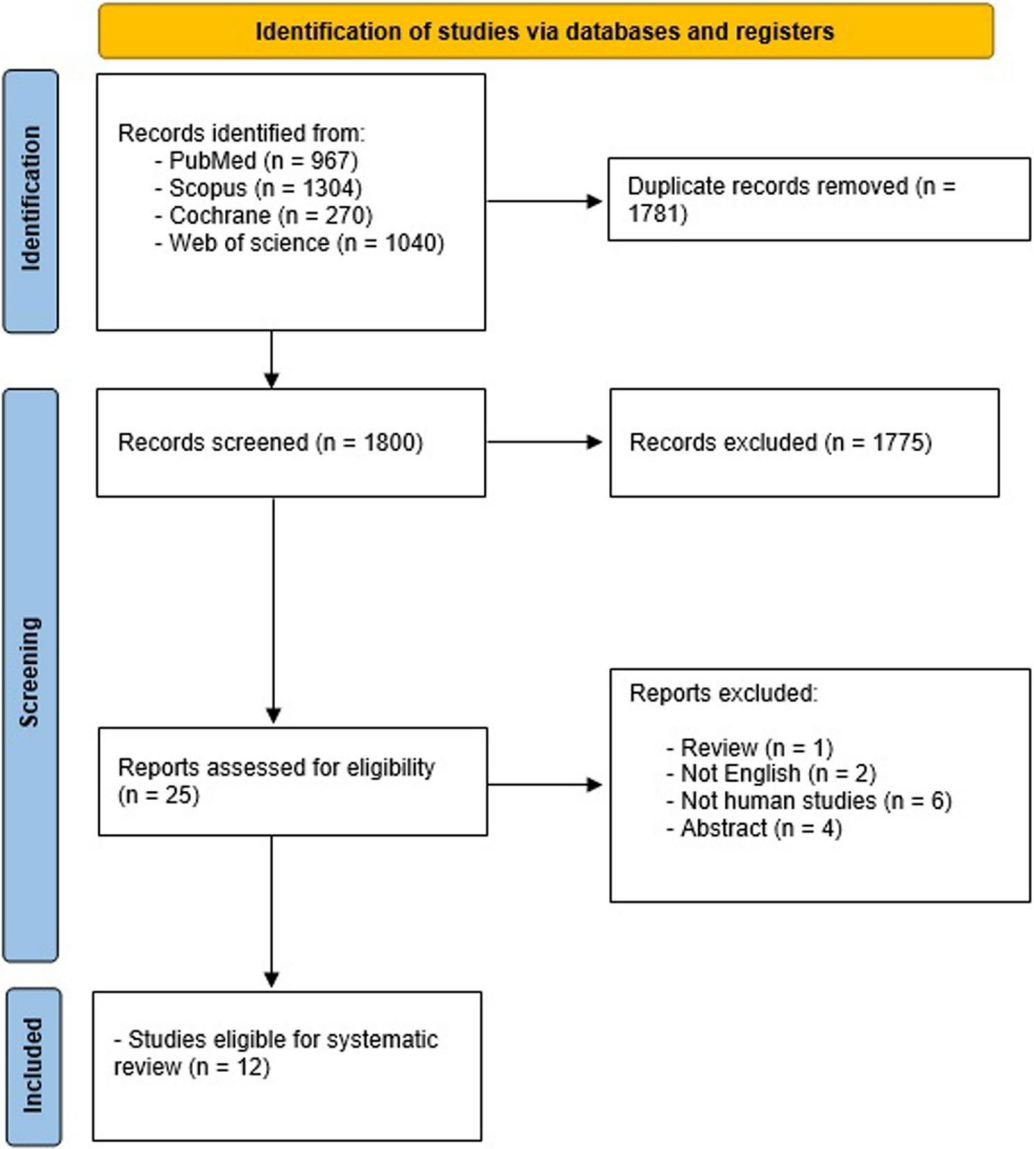
PRISMA flow chart

The initial literature search yielded 3,581 records. After employing EndNote (Clarivate Analytics, Philadelphia, United States) to remove duplicates, the dataset was narrowed down to 1,800 records for subsequent screening. During title and abstract screening, the majority of records were excluded because they did not address the prespecified PECO framework. The most common reasons for exclusion at this stage were: (i) studies that did not examine a climate-related exposure (e.g., articles on general environmental pollution, occupational chemicals, or non-climate stressors); (ii) studies whose outcomes did not concern women’s reproductive or sexual health (e.g., respiratory, cardiovascular, or general mortality outcomes); (iii) studies conducted in non-human or experimental animal models, or those using artificial heat or cold exposure; (iv) studies focused exclusively on pregnancy-specific outcomes or assisted reproductive technologies (e.g., IVF); and (v) non-original research, including narrative reviews, editorials, commentaries, and conference abstracts. A smaller proportion of records were excluded at the full-text stage because outcomes could not be disaggregated for women of reproductive age, or because exposure assessment did not include any objective or perceived climate-related variable. After this multi-step screening process, 12 observational studies were recognized [5, 6, 8, 13–21] that satisfied the inclusion criteria for the systematic review. The PRISMA flow diagram [9] provides an overview of the study selection process (Fig. 1).

### Characteristics of the included studies

The systematic review encompassed 12 observational studies, including five retrospective cohorts, one prospective cohort, and six cross-sectional studies, offering a comprehensive perspective on the link between natural climate change and reproductive and sexual health (RSH) outcomes in women. The geographic scope of the studies was extensive, encompassing diverse climate zones and spanning numerous countries worldwide. These included the USA, Hungary, Burkina Faso, Canada, Poland, India, Mongolia, Denmark, various sub-Saharan African countries, and Turkey. Notably, Barber et al. and Gray et al. surveyed an impressive number of nations, with Barber covering up to 187 countries and Gray including data from 59 countries. The primary endpoints of the studies were predominantly focused on the effect of climate change on reproductive and sexual health outcomes, including fertility rates, reproductive autonomy, women’s functionality and well-being, and ovarian reserve. This unified focus on these health outcomes provides valuable insights into the possible consequences of climate variability on women’s reproductive wellness. Table 1 outlines the key summary characteristics of the studies included in the review.

**Table 1:** Summary characteristics of the studies included in the review

Study ID	Study design	Country	Number of patients	Age (y), Mean (SD)	Inclusion criteria	Measured Outcomes	Main findings
Gray 2024 [5]	Retrospective cohort	59 low and middle-income countries	2.5 million	29.4 (9.68)	Reproductive-age women, aged 15 to 49, living in low- and middle-income countries, were incorporated based on data concerning their health status and behaviors.	Live birth, the wish for another child, employing modern contraception, being underweight (with a BMI below 18.5), and temporary migration.	Experiencing temperatures hotter than usual is linked to temporary migration, being underweight, and a lack of reproductive autonomy, although it does not show a statistically significant impact on contraception usage.
Szalma 2025 [6]	Cross-sectional	Hungary	44	32.5	The study interviewed women in Hungary aged 18 to 45 who were either without children or had only one child, encompassing those in their reproductive age.	Climate Change Perception, reproductive attitudes, employment history, and opinions on climate change and overpopulation.	The study observed a trend of considering fewer children or alternative paths like adoption due to climate change, alongside criticism towards those opting out of parenthood due to climate change impacts.
Gaskins 2021 [8]	Prospective cohort	USA	631	NR	Women aged 18–45 seeking infertility evaluation and treatment at the Massachusetts General Hospital (MGH) Fertility Center were eligible for the study, with around 60% of those contacted by research nurses enrolling.	Antral follicle count and association with climate change.	Elevated temperatures were correlated with diminished ovarian reserve, prompting concerns about accelerated reproductive aging in women due to rising temperatures worldwide.
Mahapatra 2023 [10]	Retrospective cohort	India	647	29.2(5.4)	Women aged 15–24 who have had no less than four prenatal consultations and have additionally received postnatal attention, and among women aged 15–49, those adopting hygienic menstrual protection practices and modern contraceptive methods.	Women and children’s health (WCH) that associated with climate change.	There exists a macro-level interrelation between vulnerability to climate change and Women and Children’s Health (WCH), with districts characterized by elevated climate change vulnerability showcasing deficient performance in WCH parameters.
Barber 2002 [15]	Cross-national observational study	187 countries	--	--	Countries with available data on birth rates and economic development from the World Population Data Sheet were included.	Total fertility rates for women in each country.	Total fertility rates were found to decrease significantly with increasing latitude, and fertility rates were highest in countries with average winter temperatures of around 21 °C.
Bielawska-Batorowicz 2022 [16]	Cross-sectional	Poland	104	25.6 (4.9)	Female participants who were heterosexual and had not given birth to or adopted any children, aged between 18 and 45 years old, when they joined the study.	Climate preoccupation, climate health concerns, death anxiety, and death fascination.	The study findings indicated that participants exhibited moderate levels of concern regarding climate change, including both their preoccupation with the issue and its potential health effects. Moreover, individuals with positive intentions regarding reproduction displayed reduced concern about the adverse health effects of climate change.
Carrico 2020 [17]	Retrospective cohort	Bangladesh	615	--	Females serving as heads of households or spouses, aged 11 to 23, who married between 1989 and 2013 and resided in the study area for at least one year before their first marriage.	The connection between extreme weather and the propensity for marriage.	In Bangladesh, extreme weather conditions lead to daughters marrying into poorer households with less educated husbands and also to accepting proposals from men more tolerant of intimate partner violence.
Eissler 2019 [18]	Retrospective cohort	Sub-Saharan African countries	70,879	23.8(4.7)	Data on the reproductive intentions and fertility preferences of women aged 15–49 were collected retrospectively through surveys conducted by the Demographic and Health Surveys (DHS) in sub-Saharan African nations from 1990 to 2015.	The association between climate variability and fertility intentions.	Higher-than-average temperatures correlate with women expressing a preference for smaller ideal family sizes and a lowered likelihood of desiring a first or subsequent child.
Jensen 2021 [19]	Retrospective cohort	Denmark	--	--	Analysis of historical data on human fertility trends, examining factors such as natural, anthropogenic, and infectious diseases, as well as interventions like vaccines and antibiotics. Focus on biological and socio-economic factors influencing fertility decisions over time.	Human total fertility rate with ambient temperatures.	Higher temperatures negatively impact fertility in regions experiencing monthly maximum temperatures above 15–20 °C. This underscores the significant and lasting influence of ambient temperatures on human fertility across generations.
Ozkaya 2011 [20]	Cross-sectional	Turkey	232	59.7 (9.6)	Postmenopausal females who experienced natural menopause were seen at the menopause outpatient clinic of Dr. Sami Ulus Maternity and Women’s Health Teaching and Research Hospital for standard examinations.	The age at which menopause occurs and metabolic factors in women after menopause, along with their correlation with various climates.	The Mediterranean climate correlates with premature menopause and reduced HDL levels.
Price 2019 [21]	Cross-sectional	USA	792	48.90 (17.44)	The study included women respondents from the General Social Survey (GSS) dataset who responded to questions related to concern for climate change, parenthood, education, and other relevant variables.	Levels of concern for climate change among women, the influence of education on the relationship between fertility and concern for climate change, and Differences in climate change concern between women with and without children.	Climate change concerns are similar among women with and without children. More children correlate with lower climate change concerns among women. Gender differences in climate change concerns among those with multiple children are minimal, but education complicates these findings.
Smith 2023 [22]	Cross-sectional	Canada	--	--	Young women aged 18–25, nulliparous, assigned female at birth, English-speaking, residing in British Columbia in the past 5 years.	Participants’ perspectives on climate change and childbearing by using auto-photography.	This study revealed that climate change not only impacts but actively shapes the reproductive decisions of young individuals. This suggests that climate-induced factors intertwine with feelings of eco-anxiety and socio-economic conditions, collectively influencing family planning.

### Quality assessment

According to the JBI tool [12], four cross-sectional studies were of high quality, while two studies were of moderate quality due to issues with domains. Also, most cohort studies were of moderate quality. The main reasons for downgrading studies from high to moderate quality across both cohort and cross-sectional designs were: (i) potential selection bias related to the use of non-representative or convenience samples, particularly in single-center or single-region studies; (ii) limitations in exposure measurement, given that several studies relied on ecological-level temperature data or subjective perceptions of climate change rather than individual-level objective exposure assessment; (iii) incomplete control for important confounders such as socioeconomic status, education, occupation, and access to health services; and (iv) limitations in outcome assessment, including the use of self-reported reproductive outcomes, retrospective recall, or non-standardized assessment tools. Studies retained in the moderate-quality category still met the prespecified JBI scoring thresholds and were judged to provide informative evidence, while their methodological constraints are taken into account in the interpretation of findings. More details about the criteria of this critical appraisal for cross-sectional and cohort studies were provided in Supplementary Tables 2 and 3, respectively.

### Systematic-review findings

Across the included studies, climate exposures were determined in different ways, including direct meteorological measures (temperature, seasonality, altitude) and subjective perceptions of climate change. To provide a clearer overview of the evidence base, the included studies were further synthesized by being grouped according to reproductive outcomes. Table 2 summarizes the principal findings for each category alongside details of studies and geographic settings.

**Table 2:** Summary of principal findings across categories, with corresponding studies and geographic locations

Study	Category/outcome domain	Country/setting	Climate-related exposure	Principal findings
Gray 2024 [5]	Temperature anomalies and reproductive autonomy	59 low- and middle-income countries	Temperature anomalies / unusually high temperatures	Higher temperatures were associated with reduced reproductive decision-making capacity among reproductive-age women. No consistent significant association was observed with contraceptive use.
Szalma 2025 [6]	Climate change perceptions and reproductive choices	Hungary	Perceived climate change concern	Climate change concerns influenced reproductive choices, including considering fewer children or alternative parenthood routes such as adoption. Participants also expressed social criticism toward climate-related childlessness.
Gaskins 2021 [8]	Ambient temperature and ovarian reserve	USA	Higher ambient temperature	Exposure to elevated ambient temperature was associated with reduced ovarian reserve, measured by lower antral follicle count, suggesting a possible adverse effect of heat on reproductive aging.
Mahapatra 2023 [10]	Climate vulnerability and women’s/children’s health indicators	India	District-level climate change vulnerability	Districts with higher climate vulnerability showed poorer women’s and children’s health indicators, suggesting a macro-level relationship between climate vulnerability and reproductive-health-related outcomes.
Barber 2002 [15]	Latitude, winter temperature, and fertility	187 countries	Geographic latitude and average winter temperature	Increasing latitude was associated with lower total fertility rates. The highest fertility rates were observed in countries with average winter temperatures around 21 °C, indicating a possible association between colder climates and reduced fertility.
Bielawska-Batorowicz 2022 [16]	Climate concern, death attitudes, and reproductive intentions	Poland	Perceived climate change and climate-related health concerns	Women with positive reproductive intentions reported lower concern about the negative health effects of climate change. Climate vulnerability concerns were more strongly linked to reproductive intentions than general climate concern.
Carrico 2020 [17]	Extreme weather and early marriage	Bangladesh (USA-based authors)	Heatwaves and dry spells	Moderate to severe heatwaves were associated with increased risk of marriage among girls and young women. Extreme heat appeared to act as a household coping pressure, contributing to earlier marriage and related reproductive vulnerability.
Eissler 2019 [18]	Temperature variability and reproductive intentions	Sub-Saharan African countries	Temperature and precipitation variability	Higher-than-average temperatures and climatic variability were associated with smaller ideal family size and reduced desire for additional children, especially in settings vulnerable to agricultural and socioeconomic stress.
Jensen 2021 [19]	Maximum temperature and total fertility rate	Denmark (population-level historical data)	Monthly maximum ambient temperature	Higher maximum temperatures were associated with reduced total fertility rates, particularly when monthly maximum temperatures exceeded approximately 15–20 °C. Effects extended across present and previous generations.
Ozkaya 2011 [20]	Climate region and menopausal timing	Turkey	Regional climatic zones, particularly Mediterranean climate	Living in a Mediterranean climate region during reproductive years was associated with earlier age at menopause, suggesting that regional climatic conditions may influence menopausal timing.
Price 2019 [21]	Climate change concern and family size	USA	Concern about global climate change	Greater concern about climate change was associated with smaller family size among women. Higher parity was linked with lower climate concern, possibly reflecting differences in gender norms or family-role orientation.
Smith 2023 [22]	Climate change and pregnancy intentions	Canada	Perceived climate change and eco-anxiety	Climate change influenced pregnancy intentions among young women, ranging from no perceived effect to decisions not to have children due to concerns about environmental futures and child well-being.

### Consequences of extreme temperatures on reproductive and sexual health of women

Six included studies investigated the impact of heat exposure on the RSH of women. Gray et al. [5] highlighted that unusually high temperatures are linked to reduced reproductive decision-making capabilities in women, although no significant effect was observed on contraception usage. These findings may indicate a potential link between heat exposure and women’s autonomy and health, but further research is needed. Furthermore, Gaskins et al. [17] identified a correlation between elevated temperatures and a reduced ovarian reserve measured by antral follicle count, raising concerns about potential accelerated reproductive aging globally. This evidence points towards the long-term implications of rising temperatures on female reproductive health.

Regarding high temperature and Fertility, Jensen et al. [18] reported that higher ambient temperatures negatively affect fertility, particularly in regions where monthly maximum temperatures exceed 15–20 °C. This finding illustrates the profound and enduring effects of high temperatures on human reproductive capacity across generations. This finding suggested that changes in environmental conditions may have significant impacts on human reproductive capabilities. In addition to research conducted by Carrico et al. [15] that observed the association between socioeconomic consequences with extreme weather in Bangladesh, extreme weather conditions often force daughters into marriages with less educated husbands and into households more tolerant of intimate partner violence. This highlights the complex social consequences of climatic extremes on familial and marital connections.

In terms of climatic influence on menopause, Ozkaya et al. [19] demonstrated that the Mediterranean climate is associated with premature menopause. This link provided insights into how regional climates can affect women’s reproductive health. Lastly, the study by Eissler et al. [16], which addressed family planning and temperature fluctuations, noted that higher-than-average temperatures are associated with smaller family sizes and exhibiting a reduced desire to have children; this study indicates that temperature fluctuations can significantly influence reproductive intentions and outcomes; however, causation cannot be established due to the cross-sectional design of the study.

In contrast to hot weather, a single study by Barber et al. [13] elucidated significant decreases in total fertility rates with increasing latitude, with the highest fertility rates observed in countries experiencing average winter temperatures of around 21 °C. This finding emphasizes the intricate relationship between environmental factors, such as cold weather, and their effects on women’s reproductive outcomes.

### Consequences of climate change on reproductive and sexual health of women

The impact of climate change on the RSH of women was explored in five studies. In recent research, Szalma et al. [6] observed a trend of considering fewer children or alternative paths like adoption due to climate change, alongside criticism towards those opting out of parenthood due to climate change impacts. Building upon this, Smith et al. [21] delved into the active role of climate change in shaping the reproductive decisions of young individuals, suggesting a complex interplay between climate-induced factors, eco-anxiety, and socioeconomic conditions, collectively influencing family planning processes. Furthermore, Bielawska-Batorowicz et al. [14] contributed by shedding light on participants’ moderate levels of concern regarding climate change, emphasizing their preoccupation with the issue and its potential health implications. Moreover, individuals exhibiting positive intentions towards reproduction demonstrate reduced concern regarding the adverse health effects of climate change.

Mahapatra et al. [8] uncovered a macro-level interconnection between vulnerability to climate change and Women and Children’s Health (WCH), indicating that districts with heightened climate change vulnerability exhibit deficient performance in WCH parameters, necessitating targeted interventions. Lastly, Price et al. [20] explored the nuanced dynamics of climate change concerns among women, revealing correlations between higher parity and diminished climate change concerns, with education complicating these findings.

Overall, our results indicate that extreme heat adversely impacts RSH by diminishing ovarian reserve and fertility rates. Furthermore, it influences psychosocial outcomes, driving preferences for smaller families and altering reproductive decisions, while also exacerbating socioeconomic vulnerabilities that affect RSH and autonomy.

## Discussion

### Summary of key findings

Climate change is viewed as a major risk to women’s health, particularly in their reproductive and sexual well-being. Overall, the review asserts that climate change may significantly affect women’s reproductive and sexual health. The systematic review findings underscore the profound and varied impacts of climate on female reproductive and sexual health, highlighting associations between rising temperatures and reduced fertility, accelerated reproductive aging, and altered reproductive intentions. Cold weather also demonstrates significant effects, with latitude correlating with fertility rates. Additionally, Climate variability significantly affects reproductive decisions, with climate change perceptions intersecting with family planning processes and health outcomes.

### Explanation of result findings

#### Temperature exposure and ovarian reserve

Elevated temperatures are frequently associated with lower ovarian reserve, reduced fertility rates, and changes in reproductive intentions [16–18]. Gaskins et al. (2021) reported lower antral follicle counts following sustained exposure to higher ambient temperatures, raising concern about accelerated ovarian aging. The effect was stronger when exposure was measured across three months preceding follicular assessment rather than in shorter windows, which supports the notion that chronic heat may disrupt earlier stages of follicular development [17, 22]. These findings are also consistent with other health outcomes that show stronger temperature effects in non-summer months [23–25]. The observation that women with female-factor infertility were more vulnerable suggests that pre-existing reproductive challenges may interact with environmental exposures, though such subgroup differences need replication. Nonetheless, most studies rely on observational designs and temperature metrics, which limit causal inference, but they still provide useful insights into patterns across different populations.

#### Environmental stressors and reproductive timing

According to the findings of the Barber et al. study [13], which included a cross-national analysis of 187 countries, human birth rates are lower in regions with low latitudes and during periods of low winter temperatures. This effect is substantial, given that the average birth rates in regions characterized by high latitudes and low temperatures are roughly half of those recorded in areas with low latitudes and high temperatures. This considerable effect can be attributed to variations in societal factors such as wealth, the educational level of women, the use of contraceptives, and possibly because ecological conditions become more similar to the environment of evolutionary adaptation. Another explanation of these findings might be the daily stress endured by women, leading to delayed reproductive planning. For example, in the Bolivian Altiplano, a traditional high-altitude region, women experienced delayed reproductive milestones, longer birth intervals, and a shortened reproductive span. These patterns have been linked to poor sanitation, limited nutrition, heavy workloads, and chronic hypoxia, rather than altitude alone, suggesting that the physical and daily-life stress inherent to these environments significantly shapes reproductive outcomes [26]. In contrast, milder climates may facilitate reproductive success by reducing environmental and psychosocial stressors. This pattern resembles ecological observations where reproduction in many species is timed to coincide with periods of resource abundance [27, 28]. Anthropological evidence also shows that in human populations, fertility often peaks in seasons linked to agricultural plenty and declines during periods of scarcity [29].

The shape of the graphs indicated that in extreme, high-latitude, low-temperature conditions, reproduction is suppressed to a minimum level below replacement level fertility. One hypothesis explaining the shape of the graphs is that low temperature and high latitude have a biological impact that inhibits reproduction. Possible mechanisms include alterations in ovulation rate and variations in copulation frequency [27, 28]. All of the 20 countries included in the sample with the highest latitudes currently have birth rates below the replacement fertility level. According to United Nations projections from 1998, 15 of these countries are expected to have decreased populations by 2050 (average decrease of 15.34%), while five are expected to have increased populations (average increase of 19.92%) [13].

#### Maximum temperatures and total fertility rate

Historical analyses have also linked fertility to maximum ambient temperatures. Using multi-generational data, Jensen et al. (2021) found that higher monthly maximum temperatures were associated with lower total fertility rates, particularly in regions already above 15–20 °C. The study also indicated that effects extended across generations, with parental exposures influencing the fertility of subsequent cohorts. Such findings point to climate as a long-term factor shaping demographic trends. Yet, differences in fertility trajectories across regions illustrate how socioeconomic structures, access to reproductive health services, and policy interventions can buffer or exacerbate these associations. This complexity suggests that biological sensitivity to climate is mediated by structural conditions, making cross-country comparisons essential but challenging [18].

#### Temperature anomalies and ideal family size in vulnerable regions

Reproductive intentions and family planning decisions show further evidence of the climatic influence. In sub-Saharan Africa, Eissler et al. (2019) reported that temperature and precipitation anomalies were associated with smaller ideal family sizes and a lower likelihood of desiring additional children. These effects were most pronounced in rural populations reliant on agriculture, supporting the interpretation that environmental stress reduces fertility preferences through economic insecurity [16, 30]. In contrast, survey data from the United States linked climate concern to smaller family size, though results may also reflect adherence to traditional gender norms [20]. Qualitative studies among young women in Canada and Hungary similarly noted reconsideration of childbearing considering climate change; nevertheless, reactions ranged from strong eco-anxiety to complete dismissal [6, 21]. Together, these findings suggest that both material pressures and subjective perceptions of climate shape reproductive choices [31, 32].

#### Climate change concern and family size in high-income countries

Furthermore, a cross-sectional study conducted by Price et al. [20] in the USA investigated the association between concerns about climate change and family size. There was an observed negative correlation between women’s worry about global climate change and the size of their families, suggesting that heightened environmental concerns may lead to reduced fertility rates. However, this association should not inherently result in gender disparities. An alternative interpretation of the results suggests that the decision to have more children may indicate a stronger adherence to traditional gender norms among women. Specifically, the act of having children, especially in larger numbers, may lead to a more pronounced shift in gender roles for women compared to men. This shift in roles could explain why women’s concern for climate change diminishes. This observation indicates that individuals with a higher number of children, irrespective of gender, may possess distinct characteristics compared to those with fewer children. These unmeasured differences could manifest in varying levels of concern for climate change. Their findings also suggested that women with zero to two children exhibit a greater level of concern for global climate change compared to those with more children, while the average level of concern decreases with each successive child [20].

Furthermore, Smith et al. [21] Conducted a qualitative study in Canada that utilized auto-photography and in-depth interviews to examine the influence of climate change on women’s decision-making regarding childbirth. The range of responses encompassed individuals who believed that climate change did not influence their family planning choices, alongside those who decided against having children entirely due to climate change concerns. The majority of participants expressed concerns about the future, both for themselves and for their potential children, stemming from environmental issues. The results further underscore the need for healthcare providers and decision-making resources in family planning and contraceptive use among young individuals to consider the possible impact of climate change on their decision-making process regarding pregnancy.

#### Temperature and birth rates in Low-Middle Income Countries (LMICs)

Moreover, Gray and colleagues [5] conducted a retrospective analysis across 59 low and middle-income nations. Their findings indicated that deviations in temperature lead to an increase in births and a decrease in the intention to have another child. These findings indicate that heightened temperatures may result in physiological strain, potentially impacting menstrual cycles and increasing the probability of conception during less intense heat periods. Nonetheless, the general stressful surroundings could also diminish the inclination for additional children, as families perceive less stable or more challenging living conditions for raising children. Moreover, economic and resource limitations in rural and economically disadvantaged areas and elevated temperatures can worsen living conditions by impacting agriculture and water resources. This adversity can result in increased birth rates, stemming from limited access to family planning services and diminished educational and economic prospects for women. They also discovered that deviations in temperature do not exhibit a statistically notable impact on contraceptive utilization, which could indicate varied reactions across regions, contingent on the accessibility of family planning services and cultural or economic obstacles to contraceptive access.

Similarly, Szalem and colleagues [6] conducted a qualitative investigation using semi-structured interviews carried out in Hungary. The participants comprised nulliparous women or those with one child, covering the reproductive age spectrum. The study identified a trend where individuals are considering having fewer children or exploring alternative routes, such as adoption, in response to climate change. Additionally, there was criticism directed at individuals choosing not to have children because of the impacts of climate change, underscoring the relevance of family planning. These results could stem from participants feeling a duty to reduce the effects of climate change. They similarly indicated a widespread recognition of the possible environmental consequences of having more children, which is in line with global conversations about overpopulation and sustainability.

#### Climatic region and age at natural menopause

Concerning premature menopause, Ozkaya and colleagues [19] conducted a cross-sectional study in Turkey. This study was the first to assess the relationship in the female population of Turkey, which experiences three different climates. The study indicated that the climate of the region where women spent their reproductive years was linked to the age at menopause. Women who had resided in a Mediterranean climate region exhibited the lowest average age at menopause. These results indicate that climate, in conjunction with biological and sociocultural factors, might impact menopausal timing in varying ways across regions. The reported association between Mediterranean climate and earlier menopause should be interpreted cautiously. Other large cohort data indicate that ethnicity and genetic background remain strong independent predictors of menopausal timing, for example, Latina women in the U.S. reach menopause earlier than non-Latina whites, independent of lifestyle factors [33, 34]. Further longitudinal studies are still required to investigate the net effect of climate underlying these patterns.

#### Heatwaves and the risk of early marriage

Regarding early marriage, Carrico and colleagues [15] conducted a retrospective study that integrated yearly measures of heat waves and periods of dry spells from 1989 to 2013 with survey responses from 615 women residing in southwestern Bangladesh. Their findings indicate that girls and women aged 11 to 23 faced a higher likelihood of getting married in the year of or following moderate to severe heat waves. They also discovered that the relationship between extreme heat and the probability of marriage in the same year exhibited a non-linear pattern. The likelihood of a girl or woman getting married in a particular year remained steady until heat waves lasted approximately 15 days. Beyond this threshold, there was a marked increase in the probability of marriage. Nearly one out of every four females married during years with extreme heatwaves, in contrast to 13% during years with moderate or no heatwaves (lasting 15 days or less).

Furthermore, the risk of marriage remained heightened in the year after a heatwave, indicating a delayed response in coping for certain households. This finding suggested that families with older daughters may be more inclined to use marriage as a coping strategy sooner during an environmental shock compared to those with younger daughters, who may explore alternative coping mechanisms before considering marriage. It is important to note that only heatwaves were linked to the risk of marriage. These findings underscore the intricate effects of heatwaves on marriage and reproductive health, emphasizing the necessity for multidisciplinary policies that address women’s needs.

#### Climate vulnerability concerns and reproductive intentions

Concerning Reproductive Intentions, Bielawska-Batorowicz and colleagues [14] conducted a cross-sectional study in Poland involving 104 female participants. The study’s results showed that only those with positive reproductive intentions showed a significant difference, indicating that they were less concerned about the detrimental impact of climate change on their health. The assumptions suggested that concerns about climate change and fear of death played equally significant roles as predictors of reproductive intentions. When factors significantly linked to positive reproductive intentions were integrated into the multi-factor logistic regression model, they collectively accounted for a substantial proportion of the diversity in positive intentions. Nevertheless, in this analysis, only a small number of factors retained their importance as detectors of positive reproductive intentions. Therefore, the results suggested that concerns regarding the impact of climate vulnerability on health are more significant than a broad concern about climate change.

### Consistency and inconsistency with previous findings

To date, a comprehensive systematic review dedicated to exploring the impact of climate change specific to female reproductive and sexual health remains absent from the literature. This systematic review endeavors to fill this critical gap by synthesizing existing knowledge and shedding light on the significant implications of climate change on female reproductive and sexual health.

### Robustness and limitations points

This study represents the first comprehensive review focusing on the reproductive and sexual outcomes of females in the context of climate change, providing a robust assessment of existing literature. The review encompasses studies from diverse climate zones and countries, enhancing the generalizability and relevance of the findings to global populations. However, certain limitations should be acknowledged. The primary limitation is the predominance of observational studies in the review, which may restrict the ability to establish causal relationships between climate change and reproductive health outcomes. Additionally, the majority of included studies relied on ambient temperature for exposure assessment, a method that may introduce measurement bias. It is also acknowledged that, despite AFC being used as a marker of ovarian reserve obtained via early-follicular transvaginal ultrasound and being fairly stable across cycles within skilled centers, its generalizability across settings may be constrained by inter-observer and inter-center variability, as well as by the inclusion of atretic follicles that might lead to an overestimation of functional reserve [35, 36]. Future research should consider serum biomarkers such as anti-Müllerian hormone (AMH) and correlate it with AFC, where feasible, to strengthen the ovarian reserve assessment and ameliorate reproducibility across cycles [37].

### Clinical implications and future suggestions

The study’s findings have implications for healthcare providers, informing them about the impact of climate change on reproductive health. This knowledge can help healthcare providers better educate and prepare women for potential health risks. Additionally, the results can be used to promote policies that address the underlying causes of climate change and enhance reproductive health, especially in vulnerable regions.

To address the limitations of this study, future research should focus on interventional studies to better understand the effectiveness of specific interventions in mitigating the adverse effects of climate change on reproductive health. Furthermore, there should be an emphasis on encouraging the design and inclusion of longitudinal studies to assess better causality and the long-term effects of climate change on reproductive health.

## Conclusion

This systematic review is the first systematic review that provides evidence suggesting that climate change may have important adverse effects on female reproductive and sexual health. The available observational evidence indicates that both elevated and extremely low temperatures are associated with reduced fertility rates, and that high temperatures may also influence reproductive decision-making and overall sexual well-being across diverse geographical settings. Given the cross-sectional and ecological nature of most included studies, as well as the substantial heterogeneity in exposure assessment and outcome definitions, these associations should be interpreted as suggestive rather than confirmatory of causal relationships. The findings nonetheless highlight the urgency of integrating climate considerations into public health strategies and reproductive healthcare to mitigate these potential impacts. Future research should expand on these findings through longitudinal and quasi-experimental designs that can better assess causality and the long-term consequences of climate exposure, in order to inform global health policies and interventions, such as the WHO’s Sexual and Reproductive Health Strategy and climate-health action plans, as well as interventions targeting fertility counseling, maternal health planning, and educational campaigns integrating climate resilience.

## Supplementary Information

Supplementary Material: Table 1. Search Strategy.

Supplementary Material: Table 2. Quality assessment of the included cross-sectional studies by JBI.

Supplementary Material: Table 3. Quality assessment of the included cohort studies by JBI.

## Data Availability

No datasets were generated or analysed during the current study.
