# Supplementary material for: The impacts of climate change on women’s reproductive and sexual health: a systematic review
Source: Reprod Health. 2026 May 27;23:143. doi: 10.1186/s12978-026-02375-0 (PMC13393480; doi:10.1186/s12978-026-02375-0)
Supplement: Supplementary file 1 — Supplementary Material: Table 1. Search Strategy. [file 12978_2026_2375_MOESM1_ESM.docx]

**Supplementary File 1**

**Detailed Search Strategy**

*Manuscript: "The Impacts of Climate Change on Women's Reproductive and Sexual Health: A Systematic Review"*

*Journal: Reproductive Health*

*PROSPERO registration: CRD42024575251*

# 1. Overview of the search strategy

A comprehensive, pre-piloted search strategy was designed to ensure thorough and unbiased coverage of the literature on the impact of climate change on women's reproductive and sexual health. The strategy was structured around the PECO framework (Population, Exposure, Comparator, Outcome) defined in the main manuscript and applied consistently across four bibliographic databases: MEDLINE/PubMed, Scopus, Web of Science, and the Cochrane Library. The final search was conducted up to May 2024. Search terms combined controlled vocabulary (Medical Subject Headings [MeSH] in PubMed and equivalent indexing terms where available in other databases) with free-text terms in titles, abstracts, and keywords, using Boolean operators (AND/OR) and truncation (*) where supported. The search strategy was pilot-tested for sensitivity and specificity against a set of pre-identified benchmark studies prior to full deployment.

In addition to database searching, the reference lists of all included studies and of relevant systematic and narrative reviews were hand-searched (backward citation tracking). Forward citation tracking was performed for key included articles using the "cited by" function in Scopus and Web of Science. Grey literature from international organizations (WHO, UNFPA, IPCC) and government climate–health reports was consulted informally to contextualize the findings during the discussion stage; this grey literature was not included in the quantitative or qualitative synthesis.

# 2. PECO framework and key concepts

Table S1.1 summarizes the PECO framework that guided the construction of the search strategy and the selection of search terms across all databases.

***Table S1.1. PECO framework used to derive the search concepts and terms.***

| **PECO element** | **Definition** | **Key concepts used in the search** |
| --- | --- | --- |
| **Population (P)** | Women of reproductive age (15–49 years), and women approaching or undergoing menopause. | women; female; reproductive age; menopause; perimenopause. |
| **Exposure (E)** | Climate change and climate-related variables (objective meteorological measures and subjective perceptions of climate change). | climate change; global warming; ambient temperature; heat exposure; heatwave; extreme weather; environmental temperature; seasonality; latitude; climate variability; climate perception; eco-anxiety. |
| **Comparator (C)** | No formal comparator was required, given the observational and ecological nature of the included studies. | — (not applied as a search filter) |
| **Outcomes (O)** | Reproductive and sexual health outcomes in women, including biological/physiological and psychosocial/behavioral outcomes. | reproductive health; sexual health; fertility; ovarian reserve; antral follicle count; AMH; menopause timing; age at menopause; reproductive decision-making; reproductive intention; family planning; childbearing; contraception; eco-anxiety; forced marriage; early marriage. |

# 3. Bibliographic databases, dates, and filters

Table S1.2 summarizes the databases searched, the interface used, the date of the last search, and the filters or limits applied. No date restriction was applied at the indexing level; the only language restriction applied was English, in line with the predefined inclusion criteria.

***Table S1.2. Databases, interfaces, search dates, and filters applied.***

| **Database** | **Interface / Platform** | **Date of last search** | **Filters / Limits applied** |
| --- | --- | --- | --- |
| **MEDLINE / PubMed** | NCBI PubMed | May 2024 | English; Humans; no date restriction at indexing level. |
| **Scopus** | Elsevier Scopus | May 2024 | English; Article / Review (document type); no date restriction. |
| **Web of Science** | Clarivate Web of Science Core Collection | May 2024 | English; Article / Review (document type); no date restriction. |
| **Cochrane Library** | Wiley – Cochrane Central Register of Controlled Trials (CENTRAL) and Cochrane Database of Systematic Reviews (CDSR) | May 2024 | English; no date restriction. |

# 4. MEDLINE / PubMed search strategy

The PubMed search combined MeSH terms ([MeSH]) and free-text terms searched in title and abstract ([tiab]). Truncation (*) was used to capture variant word endings (e.g., reproductive intention* retrieves intention and intentions). The final search line combined the three concept blocks (Exposure, Population, Outcomes) with the Boolean AND operator, and standard PubMed filters (English language; Humans) were applied at the final stage. The full PubMed strategy is reproduced in Table S1.3.

***Table S1.3. PubMed search lines and number of records retrieved (last search: May 2024).***

| **#** | **Concept** | **PubMed search line** | **Results (n)** |
| --- | --- | --- | --- |
| **#1** | Exposure: Climate change | ("Climate Change"[MeSH] OR "Global Warming"[MeSH] OR "Hot Temperature"[MeSH] OR "Extreme Heat"[MeSH] OR "Weather"[MeSH] OR "Seasons"[MeSH] OR "climate change"[tiab] OR "global warming"[tiab] OR "ambient temperature"[tiab] OR "heat exposure"[tiab] OR "heat wave*"[tiab] OR "heatwave*"[tiab] OR "extreme temperature*"[tiab] OR "extreme weather"[tiab] OR "environmental temperature"[tiab] OR "climate variability"[tiab] OR "climate perception*"[tiab] OR "eco-anxiety"[tiab] OR "climate anxiety"[tiab]) | — |
| **#2** | Population: Women | ("Women"[MeSH] OR "Women's Health"[MeSH] OR "Female"[MeSH] OR women[tiab] OR woman[tiab] OR female*[tiab] OR girls[tiab]) | — |
| **#3** | Outcomes: Reproductive & sexual health | ("Reproductive Health"[MeSH] OR "Sexual Health"[MeSH] OR "Fertility"[MeSH] OR "Infertility, Female"[MeSH] OR "Ovarian Reserve"[MeSH] OR "Menopause"[MeSH] OR "Menopause, Premature"[MeSH] OR "Family Planning Services"[MeSH] OR "Birth Rate"[MeSH] OR "Reproductive Behavior"[MeSH] OR "Contraception"[MeSH] OR "reproductive health"[tiab] OR "sexual health"[tiab] OR fertility[tiab] OR "ovarian reserve"[tiab] OR "antral follicle count"[tiab] OR "anti-Mullerian hormone"[tiab] OR AMH[tiab] OR menopause[tiab] OR "age at menopause"[tiab] OR "reproductive decision*"[tiab] OR "reproductive intention*"[tiab] OR "family planning"[tiab] OR "birth rate*"[tiab] OR "fertility rate*"[tiab] OR childbearing[tiab] OR contraception[tiab] OR "forced marriage"[tiab] OR "early marriage"[tiab]) | — |
| **#4** | **Combined: #1 AND #2 AND #3** | **#1 AND #2 AND #3** | **1,184** |
| **#5** | Filters applied | #4 AND English[lang] AND Humans[Filter] | 1,036 |

# 5. Scopus search strategy

The Scopus search used the TITLE-ABS-KEY field tag, which simultaneously searches article titles, abstracts, and author/index keywords. The same three-concept structure (Exposure × Population × Outcomes) was applied. Document type was restricted to Article and Review, and language to English, using the LIMIT-TO operator. The full Scopus strategy is presented in Table S1.4.

***Table S1.4. Scopus search lines and number of records retrieved (last search: May 2024).***

| **#** | **Concept** | **Scopus search line** | **Results (n)** |
| --- | --- | --- | --- |
| **#1** | Exposure | TITLE-ABS-KEY ( "climate change" OR "global warming" OR "ambient temperature" OR "heat exposure" OR "heat wave*" OR "heatwave*" OR "extreme temperature*" OR "extreme weather" OR "environmental temperature" OR "climate variability" OR "climate perception*" OR "eco-anxiety" OR "climate anxiety" ) | — |
| **#2** | Population | TITLE-ABS-KEY ( women OR woman OR female* OR girls ) | — |
| **#3** | Outcomes | TITLE-ABS-KEY ( "reproductive health" OR "sexual health" OR fertility OR "ovarian reserve" OR "antral follicle count" OR "anti-Mullerian hormone" OR AMH OR menopause OR "age at menopause" OR "reproductive decision*" OR "reproductive intention*" OR "family planning" OR "birth rate*" OR "fertility rate*" OR childbearing OR contraception OR "forced marriage" OR "early marriage" ) | — |
| **#4** | **Combined** | **#1 AND #2 AND #3 AND ( LIMIT-TO ( LANGUAGE,"English" ) ) AND ( LIMIT-TO ( DOCTYPE,"ar" ) OR LIMIT-TO ( DOCTYPE,"re" ) )** | **1,278** |

# 6. Web of Science Core Collection search strategy

The Web of Science search used the Topic (TS) field, which searches title, abstract, author keywords, and Keywords Plus®. The same three-concept structure was applied; results were refined by Language = English and Document Type = Article or Review Article. The full Web of Science strategy is presented in Table S1.5.

***Table S1.5. Web of Science search lines and number of records retrieved (last search: May 2024).***

| **#** | **Concept** | **Web of Science search line (Topic = TS)** | **Results (n)** |
| --- | --- | --- | --- |
| **#1** | Exposure | TS = ("climate change" OR "global warming" OR "ambient temperature" OR "heat exposure" OR "heat wave*" OR "heatwave*" OR "extreme temperature*" OR "extreme weather" OR "environmental temperature" OR "climate variability" OR "climate perception*" OR "eco-anxiety" OR "climate anxiety") | — |
| **#2** | Population | TS = (women OR woman OR female* OR girls) | — |
| **#3** | Outcomes | TS = ("reproductive health" OR "sexual health" OR fertility OR "ovarian reserve" OR "antral follicle count" OR "anti-Mullerian hormone" OR AMH OR menopause OR "age at menopause" OR "reproductive decision*" OR "reproductive intention*" OR "family planning" OR "birth rate*" OR "fertility rate*" OR childbearing OR contraception OR "forced marriage" OR "early marriage") | — |
| **#4** | **Combined + filters** | **#1 AND #2 AND #3 Refine by: Languages = English; Document types = Article OR Review Article** | **1,063** |

# 7. Cochrane Library search strategy

The Cochrane Library (CENTRAL and CDSR) was searched using free-text terms in title, abstract, and keyword fields (:ti,ab,kw). The strategy was simplified relative to the major bibliographic databases because the Cochrane Library indexes a more focused set of interventional and systematic-review records. The full Cochrane Library strategy is presented in Table S1.6.

***Table S1.6. Cochrane Library search lines and number of records retrieved (last search: May 2024).***

| **#** | **Concept** | **Cochrane Library search line** | **Results (n)** |
| --- | --- | --- | --- |
| **#1** | Exposure | ("climate change" OR "global warming" OR "ambient temperature" OR "heat exposure" OR "heat wave*" OR "heatwave*" OR "extreme temperature*" OR "extreme weather" OR "climate variability" OR "eco-anxiety"):ti,ab,kw | — |
| **#2** | Population | (women OR woman OR female* OR girls):ti,ab,kw | — |
| **#3** | Outcomes | ("reproductive health" OR "sexual health" OR fertility OR "ovarian reserve" OR menopause OR "family planning" OR "birth rate*" OR "fertility rate*" OR contraception):ti,ab,kw | — |
| **#4** | **Combined** | **#1 AND #2 AND #3** | **56** |

# 8. Summary of records retrieved

Table S1.7 summarizes the number of records retrieved from each database, together with the number of additional records identified through hand-searching, the total number of records identified prior to duplicate removal, the number of records remaining after duplicate removal in EndNote, and the final number of studies included in the systematic review. The corresponding PRISMA 2020 flow diagram is shown as Figure 1 in the main manuscript.

***Table S1.7. Summary of records identified across databases and hand-searching.***

| **Database** | **Date of search** | **Records retrieved** |
| --- | --- | --- |
| **MEDLINE / PubMed** | May 2024 | 967 |
| **Scopus** | May 2024 | 1,304 |
| **Web of Science** | May 2024 | 1,040 |
| **Cochrane Library** | May 2024 | 270 |
| **Total records identified** |  | **3,581** |
| **After duplicate removal (EndNote)** |  | **1,800** |
| **Final number of studies included** |  | **12** |

# 9. Notes and limitations of the search

**Pilot testing.** The search strategy was pilot-tested against a set of pre-identified benchmark studies relevant to climate change and women's reproductive and sexual health. Adjustments to the free-text and MeSH term combinations were made until the pilot returned all benchmark studies, confirming the sensitivity of the strategy.

**Translation across databases.** The PubMed master strategy was systematically translated to Scopus, Web of Science, and the Cochrane Library using interface-specific syntax (TITLE-ABS-KEY, TS, and :ti,ab,kw fields, respectively) while preserving the underlying Boolean structure and the three-concept design (Exposure × Population × Outcomes).

**Language restriction.** Only studies published in English were eligible, in line with the predefined inclusion criteria of the review. This restriction is acknowledged in the Limitations section of the main manuscript as a potential source of language and publication bias.

**Date of last search.** The final search across all four databases was completed in May 2024. No automated alerts were used to update the search after this date.

**Duplicate removal.** All retrieved records were exported to EndNote (Clarivate Analytics, Philadelphia, PA, USA) and de-duplicated using the built-in duplicate detection tool, with manual verification of borderline cases.

**Screening.** Title and abstract screening, full-text eligibility assessment, data extraction, and quality appraisal were performed independently by two reviewers using the Rayyan web application, with disagreements resolved through discussion and, where consensus could not be reached, by consultation with a third senior researcher (see Methods – Screening procedure in the main manuscript).
