# Supplementary material for: The impacts of climate change on women’s reproductive and sexual health: a systematic review
Source: Reprod Health. 2026 May 27;23:143. doi: 10.1186/s12978-026-02375-0 (PMC13393480; doi:10.1186/s12978-026-02375-0)
Supplement: Supplementary file 2 — Supplementary Material: Table 2. Quality assessment of the included cross-sectional studies by JBI. [file 12978_2026_2375_MOESM2_ESM.docx]

| **Study ID** | **Were the criteria for inclusion in the sample clearly defined?** | **Were the study subjects and the setting described in detail?** | **Was the exposure measured in a valid and reliable way?** | **Were objective, standard criteria used for measurement of the condition?** | **Were confounding factors identified?** | **Were strategies to deal with confounding factors stated?** | **Were the outcomes measured in a valid and reliable way?** | **Was appropriate statistical analysis used?** | **Total score** |
| --- | --- | --- | --- | --- | --- | --- | --- | --- | --- |
| **Szalma 2025** | **Yes** | **Yes** | **No** | **Yes** | **No** | **No** | **Yes** | **Yes** | **5** |
| **Smith 2023** | **Yes** | **Yes** | **No** | **Yes** | **No** | **No** | **Yes** | **Yes** | **5** |
| **Bielawska-Batorowicz 2022** | **Yes** | **Yes** | **Yes** | **Yes** | **No** | **No** | **Yes** | **Yes** | **6** |
| **Price 2019** | **Yes** | **Yes** | **Yes** | **Yes** | **Yes** | **Yes** | **Yes** | **Yes** | **8** |
| **Ozkaya 2011** | **Yes** | **Yes** | **Yes** | **Yes** | **No** | **No** | **Yes** | **Yes** | **6** |
| **Barber 2002** | **Yes** | **Yes** | **Yes** | **Yes** | **Yes** | **Yes** | **Yes** | **Yes** | **8** |

**Supplementary Table 2:** Quality assessment of the included cross-sectional studies by JBI
