# Supplementary material for: The impacts of climate change on women’s reproductive and sexual health: a systematic review
Source: Reprod Health. 2026 May 27;23:143. doi: 10.1186/s12978-026-02375-0 (PMC13393480; doi:10.1186/s12978-026-02375-0)
Supplement: Supplementary file 3 — Supplementary Material: Table 3. Quality assessment of the included cohort studies by JBI. [file 12978_2026_2375_MOESM3_ESM.docx]

| **Study ID** | **Were the two groups similar and recruited from the same population?** | **Were the exposures measured similarly to assign people to both exposed and unexposed groups?** | **Was the exposure measured in a valid and reliable way?** | **Were confounding factors identified?** | **Were strategies to deal with confounding factors stated?** | **Were the groups/participants free of the outcome at the start of the study (or at the moment of exposure)?** | **Were the outcomes measured in a valid and reliable way?** | **Was the follow-up time reported sufficient to be long enough for outcomes to occur?** | **Was the follow-up complete, and if not, were the reasons for loss of follow-up described and explored?** | **Were strategies to address incomplete follow-up utilized?** | **Was appropriate statistical analysis used?** | **Total score** |
| --- | --- | --- | --- | --- | --- | --- | --- | --- | --- | --- | --- | --- |
| **Gray 2024** | **NA** | **NA** | **Yes** | **Yes** | **Yes** | **Yes** | **Yes** | **Yes** | **Yes** | **Yes** | **Yes** | **9** |
| **Mahapatra 2023** | **NA** | **NA** | **Yes** | **No** | **No** | **Yes** | **Yes** | **NA** | **Yes** | **Yes** | **Yes** | **6** |
| **Gaskins 2021** | **NA** | **NA** | **Yes** | **Yes** | **Yes** | **Yes** | **Yes** | **Yes** | **No** | **No** | **Yes** | **7** |
| **Jensen 2021** | **NA** | **NA** | **Yes** | **Yes** | **Yes** | **NA** | **Yes** | **yes** | **NA** | **NA** | **Yes** | **6** |
| **Carrico 2020** | **NA** | **NA** | **Yes** | **Yes** | **Yes** | **Yes** | **Yes** | **NA** | **NA** | **Yes** | **Yes** | **7** |
| **Eissler 2019** | **NA** | **NA** | **Yes** | **Yes** | **Yes** | **Yes** | **Yes** | **NA** | **NA** | **NA** | **Yes** | **6** |

**Supplementary Table 3:** Quality assessment of the included cohort studies by JBI
